# Storage and Export of Atmospheric Hg, Pb, Al, and Fine Particulate Matter (PM2.5) from Forest Trees

**DOI:** 10.1021/acs.est.5c11154

**Published:** 2025-11-04

**Authors:** Joshua D. Landis

**Affiliations:** Department of Earth and Planetary Sciences, 3728 Dartmouth College, 19 Fayerweather Hill Road, Hanover, New Hampshire 03755, United States

**Keywords:** chronometer, canopy, forest, phyllosphere, PM2.5, mercury (Hg), lead (Pb), FPOM

## Abstract

A mass balance of whole-tree inventories and fluxes in throughfall and litterfall reveals that mature forest trees strongly retain atmospheric deposition of fine particulate matter (PM2.5) and metals, including Hg, Pb, and Al, and store this material over decadal time scales. Wet and dry deposition are retained with similar efficiencies (56 and 52% of totals, respectively). Accumulation of Hg by direct absorption of gaseous elemental mercury (GEM) contrasts with crustal metals (e.g., Al) that are likely to be resuspended from the surrounding ecosystem. Due to long storage times and nonsteady state with respect to declining industrial emissions, long-lived nonfoliar tissues of the canopy, including lichen, moss, mold, bark, and arboreal soil (hereafter phyllosphere), may store 10–100 times the contemporary fluxes of Hg and Pb as a legacy of past emissions. Release of stored legacy Hg and Pb is mediated by dissolved organic carbon (DOC) and fine particulate matter (FPOM) even as new deposition continues to be absorbed in complex exchange within the phyllosphere. These results advance a new understanding of metal biogeochemical cycling in forest canopies that includes substantial storage, exchange, and release over decadal time scales.

## Graphical abstract

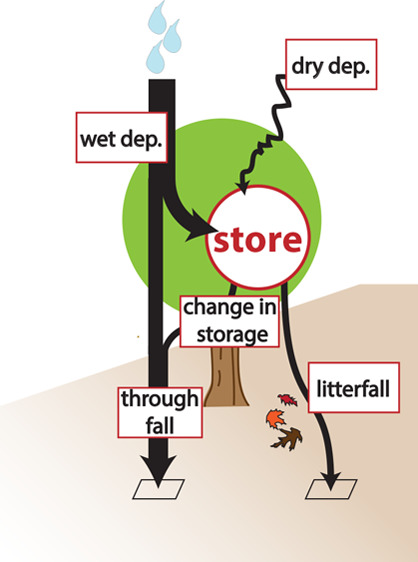

## 1 Introduction

Above-ground biomass in terrestrial ecosystems represents a vast reservoir of natural surfaces that intercepts and stores large quantities of atmospherically deposited metals.^1–4^ Although conventional ecosystem mass balances include fluxes from the canopy in both litterfall and throughfall (precipitation passing through the canopy), the contributions of long-lived ecosystem components such as bark, moss, lichen, fungus, mold, vascular epiphytes, and arboreal soils^5^ that develop from this organic matter, to the absorption of metals from atmospheric deposition remain largely unconstrained (hereafter, we refer to nonfoliar canopy components as phyllosphere). Moreover, the time scales over which metals sequestered in the phyllosphere are resuspended to the atmosphere as fine particulate matter (PM2.5, PM10) or transferred to underlying soils are generally unknown. Phyllosphere dynamics are especially important for understanding biogeochemical cycling of anthropogenic metals (e.g., Pb and Hg) and other pollutants since the phyllosphere is unlikely to be at steady-state, e.g., in cases of historic changes in atmospheric emissions. Pb and Hg atmospheric concentrations, for example, have responded to increasingly stringent regulatory constraints following decades of industrial emissions. Nonetheless, even as atmospheric fluxes have declined in the northeastern United States over decades since peaking in the 1980s–1990s, metals from legacy deposition might contribute to contemporary atmospheric, throughfall, and litterfall fluxes.^6–9^ Understanding the full impacts of historic pollutant deposition and accurately assessing contemporary metal biogeochemical cycles requires an improved understanding of metal storage in the forest canopy.

Concerns over the atmospheric dispersion of toxic metals began with nuclear fission products and radioactive aerosols produced during atmospheric bomb-testing,^10–14^ and soon thereafter included heavy metals from automotive and industrial emissions,^15,16^ which were also closely tied to the problem of acid rain.^17,18^ More recently, the accumulation of atmospheric mercury (Hg) in terrestrial ecosystems is a burgeoning priority due to its high toxicity and propensity for bioaccumulation in both terrestrial and aquatic food webs.^19,20^ On the order of ten times the annual flux of Hg in precipitation and litterfall may be stored in the phyllosphere, calling into question both the true scale of deposition as well as the longevity of Hg in the canopy ecosystem.^2–4,6^ It is already understood that epiphytic mosses and lichens are efficient sinks for atmospheric pollutants to such an extent that they are used as biomonitors of metal deposition and ecosystem health.^21–25^ Notably, gasoline Pb persists in contemporary PM decades after its elimination from automotive use, from resuspension of contaminated soil^8,26,27^ or biogenic dust from the phyllosphere.^1^ Throughfall, which is precipitation passing through the forest canopy, is strongly enriched in metals derived from storage in the canopy.^28^ While the phyllosphere represents a reservoir of long-term PM and metal storage that likely contributes to ongoing throughfall fluxes to underlying soils, quantifying the contributions of the phyllosphere to pollutant budgets and exposure pathways remains an outstanding challenge. This is due specifically to the unknown age or lifetime of these perennial materials: without knowing the time scales over which they accumulate metals, it is not possible to calculate equivalent fluxes from the atmosphere.

Advances in the use of fallout radionuclide (FRN) chronometry can now measure time scales and fluxes of metal accumulation in terrestrial ecosystems. FRN chronometry is used to measure rates of atmospheric pollutant and carbon accumulation in soils,^6,29^ as well as in fruticose lichens.^24^ The half-times of natural ^7^Be and ^210^Pb in vegetation with respect to their physicochemical removal (or weathering) are >900 days, which demonstrates permanent sorption of particle-reactive atmospheric metals to foliage^30^ (their radioactive half-lives are 54 days and 22.3 years, respectively). Similar half-times are observed for radioactive ^137^Cs released from the tragic Fukushima Daichi nuclear power accident into surrounding forests^31^ and experimental applications of Hg isotope spikes to entire watersheds.^32^ Long weathering half-times for FRNs and metals demonstrate their irreversible binding to natural organic matter (NOM) surfaces, to be subsequently weathered as PM from phyllosphere reservoirs.^33^ In a companion study of FRNs and metals in throughfall, we found that the canopy is a net sink for FRNs (throughfall enrichment factors <1) but a net source for most other metals, including Hg, Pb, and Be, due to a release or change in storage (Δ*S*) from the phyllosphere (enrichment factors 2–5).^28^ Δ*S* represents a release from long-term storage, which is distinct from contemporary dry deposition, and is facilitated by the transformation and association of metals with DOC and FPOM in a close coupling of metal, carbon, and hydrologic cycles.^28^ We estimated that Δ*S* contributes 29, 40, 63, and 21%, respectively, of ^210^Pb, Hg, Pb, and Al export from the canopy in throughfall.^28^

While the phyllosphere is likely to be a significant source of PM and metals to underlying ecosystems, the magnitude and time scale of its PM and pollutant storage remain largely unknown. To address this knowledge gap, here, we apply FRN chronometry to quantify the accumulation, storage, export, and residence times of atmospheric PM metals in the phyllosphere by constructing a mass balance of flows of PM and associated metals through the phyllosphere. This present study adds measurements of FRNs and major/trace elements (MTEs), including Hg, Pb, and Al from a whole-tree harvest and litterfall to data sets of wet and dry atmospheric deposition,^33,34^ throughfall,^28^ and soil measurements^6,35^ that are described elsewhere.

## 2 Methods

Immediately prior to annual autumn leaf fall in October 2021, a 68-year-old, 66-foot-tall red oak (diameter at breast height, dbh = 16 cm) with a crown diameter of ca. 2 m was felled by chainsaw from the forest canopy at the Beaver Meadow site in the village of Sharon, Vermont, in the northeastern US. This is a small rural village (population ca. 1500) characterized by low-density development and unpaved roads. The tree was immediately trimmed, limbed, and bucked into 54 sections and then transported to the laboratory for further analysis (Figures 1 and S1). All foliage and tree sections were weighed and dimensioned (length, diameter, outer surface area) to produce whole-tree mass balances for ^7^Be, ^210^Pb, and trace metals Hg, Pb, and Al. We measured only outermost surfaces presumed to have no active metabolic contributions from the tree (absent inner bark, cambium, and bole wood). Twigs were trimmed from all branches, and branches were separated into <1, 1–2, and 2–3 cm diameters by pruning. Leaves were collected in five tiers along the height of the tree (Figure 1). The age of the tree was estimated to be 68 years by counting annual rings at the trunk base. It is difficult to estimate the volume of particulate matter that may have been dislodged from the tree during felling, and while this fraction did not appear significant relative to the material retained by the tree, the reported metal inventories in the phyllosphere may therefore be seen as minimum estimates.

**Figure 1:**
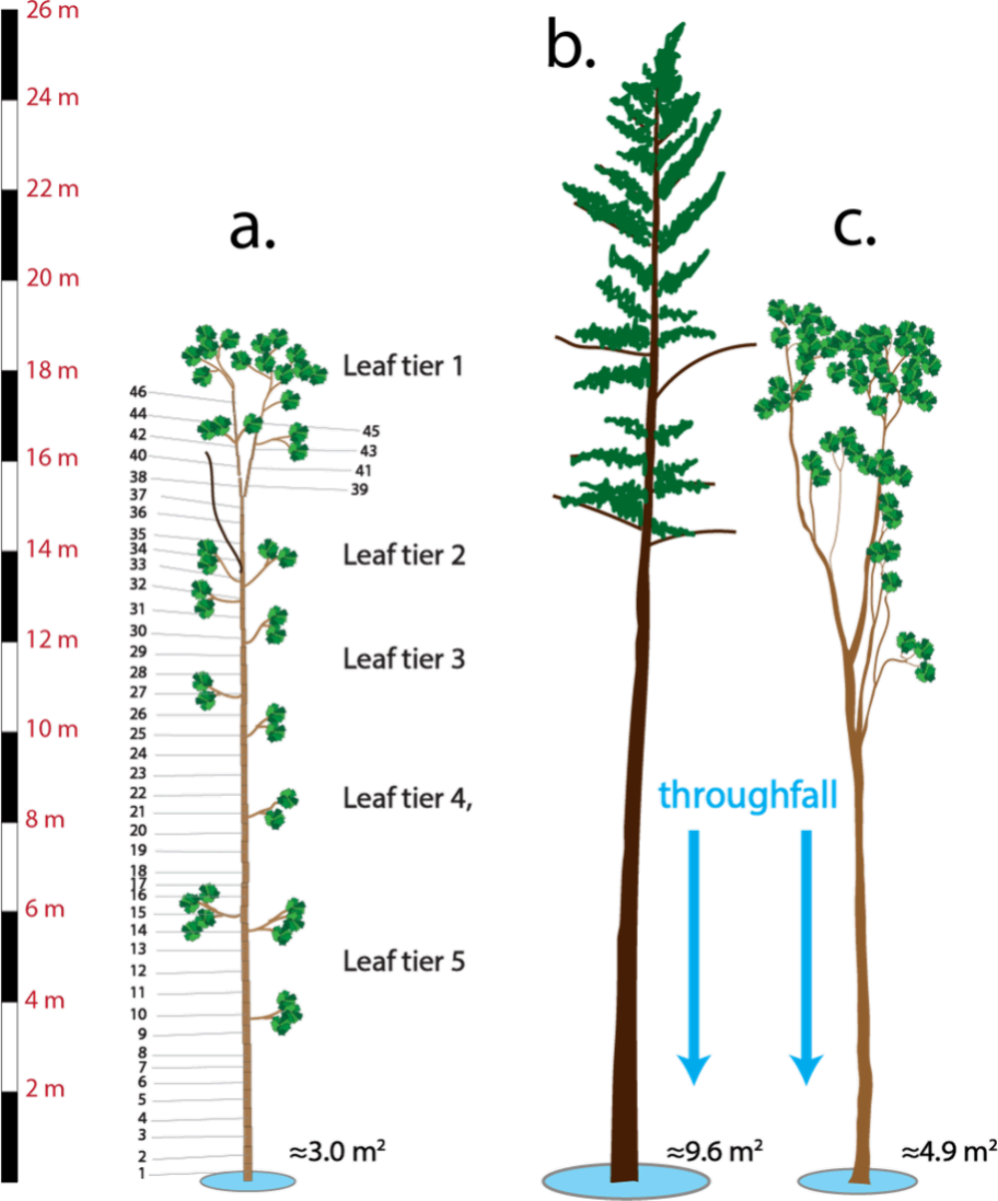
Schematic of whole-tree mass balance, and two adjacent trees where throughfall/litterfall was measured.^28^ (a) Whole-tree red oak with measured sections numbered as indicated, leaf area index (LAI) = 3.0 and crown projected area = 3.0 m^2^, (b) trees studied in throughfall/litterfall, including eastern white pine, LAI = 2.8, area = 9.6 m^2^, (c) red oak, LAI = 3.1, area = 4.9 m^2^.^28^ Tree silhouettes were traced from the photo.

Fifteen representative sections from the bottom, middle, upper, and crown positions were selected for FRN and metals analysis, and results were scaled to the whole tree using measured surface area dimensions. The short half-life of ^7^Be requires all samples to be measured within approximately one month of collection, which limits the number of samples that can be measured. Distinct tissue types on the tree surface were separated as follows (Figure S2). Whole live moss and fruticose lichen were trimmed with a stainless scalpel. Surficial moss, fruticose and crustose lichen, mold, and arboreal soil (hereafter called the live surface) were removed by gently scraping the surface of the bark with the scalpel edge (young red oak has smooth bark). Finally, the outermost ∼0.5 mm was shaved with a scalpel to recover the dead bark surface (Figure S3). Twigs and branches were similarly represented by shaving the outermost bark.^28^

Fresh litterfall (LF) was collected approximately biweekly or monthly during the autumn litterfall of 2020 and approximately seasonally through the full year during 2021–2023, using both 0.2 m^2^ mesh baskets and 10 m^2^ landscaping fabric as collection surfaces. The fabric is a multilayer polypropylene mesh with a matrix grid pattern that allows free drainage of precipitation to minimize the absorption of new FRNs and metals in wet deposition to collected litter. A large area of fabric is required to collect representative fine particulate matter (FPOM) in litterfall in gram quantities sufficient for analysis. Adjacent soils were collected to assess the steady-state FRN and total metal inventories. Continuous long-term bulk atmospheric deposition measurements were recorded at the nearby Shattuck Observatory (15 km away) as previously described.^28,33,34^ A long-term timeseries of foliage from red oak at Shattuck Observatory is also included here as originally described by Landis et al.^30^ Leaves were sampled approximately monthly throughout the year, and we note that, while red oak is deciduous, it retains dead leaves on branches in its canopy positions through winter and into its second spring (a phenomenon called marcescence).

Throughfall (TF) and paired openfall fluxes of FRNs and MTEs were measured at the Beaver Meadow site in event-based sampling for 52 storm events in 2020–2022 and are described elsewhere.^28^ Throughfall was collected under mature trees of eastern white pine (*Pinus strobus*; dbh = 59 cm) and eastern red oak (dbh = 28 cm) within 50 m of the felled whole-tree oak (Figure 1), and these trees are represented in the canopy mass balance of Section 4. Atmospheric fluxes are used to establish the steady-state FRN and total MTE inventories for comparison with the inventories recovered in the whole-tree compartments.

FRNs were measured in bulk samples by gamma spectrometry as described previously, where quality control has been shown to provide percent-level precision with reference materials IAEA385, IAEA447, CCRMP CLV1, and NIST 4353a.^36,37^ Briefly, bulk samples are packed in 10 or 110 cm^3^ Petri dishes and measured in close-in geometry directly on the detector end-cap. Calibration was performed using matrix-matched standards doped with 1% w/w uranium ore (CCRMP BL-5) or thorium ore (CCRPM OKA-2), and a U-ore point source was used to correct self-attenuation at all energies.^37^ Bulk Hg was measured by combustion atomic absorption (direct mercury analyzer: Milestone DMA-80) with NIST1547 (peach leaves) and NIST 2706 (New Jersey soil) reference materials, running each 10 samples for quality control. Recovery of Hg in these standards was 99.3 ± 8.6% at ∼1 ng and 103.4 ± 4.8% at ∼5 ng^6^. Other metals were measured in aqua regia acid extractions by ICPOES (Spectro ARCOS in radial mode) to provide operationally defined soluble metal fractions. Approximately 2 g of powdered sample was extracted in reverse aqua regia (9:3 mL HNO_3_:HCl).^28^ Water reference materials NIST1640a and 1643f were used as instrumental checks with recoveries of metals in solution concentrations of 1–10 ng g^–1^ as follows: Al = 100.7 ± 2.8%, As, 97.4 ± 19.9%, Ba = 101.6 ± 1.3%, Be = 104.4 ± 2.1%, Ca = 101.3 ± 1.3%, Cd = 103.7 ± 2.9%, Co = 97.5 ± 3.3%, Cr = 101.7 ± 6.2%, Fe = 104.9 ± 1.8%, Ni = 98.8 ± 3.5%, Pb = 105 ± 9.9%, V = 93.0 ± 6.1%, Zn = 112.2 ± 6.9%.

Whole tree inventories of atmospheric metals were calculated by the multiplication of measured concentrations with sample masses for all leaves, twigs, branches, and 6–15 representative tree sections. Masses were scaled to the whole tree by surface area, as calculated from measured length and diameter dimensions of each tree section. Inventories in the whole-tree oak were scaled to larger throughfall trees using standard allometric equations.^38^ All reported inventories are normalized to the projected area of the tree crown (mass or activity per m^2^ projected area) as follows:

$$\mathrm{total}\;[\mathrm{Bq}\;\mathrm{or}\;\mu g\;m^{-2}]=\frac{1}{A_{c}}\sum_{n}\sum_{i}C_{R}m_{R}\frac{{\mathrm{SA}}_{i}}{{\mathrm{SA}}_{R}}$$ 1

where *A*_c_ is the projected area of the tree crown, *C*_R_ is the concentration of the metal in the reference section, *m*_R_ is the total mass of the material collected from the reference section, and SA is the surface area of the reference and each of 46 modeled tree sections (*i*) for 7 tree surfaces (*n*).

Uncertainties (1σ) for annual open, throughfall, and Δ*S* fluxes are propagated from analytical uncertainties, and, due to the large number of measurements (>10^2^), fall in the range of 2–5%. Uncertainties on dry deposition are reported as standard errors from model parameter predictions,^28^ and litterfall uncertainties are reported as standard errors of multiyear averages. Mass balance uncertainties are estimated by propagation from these sources.

## 3 Results and Discussion

### 3.1 Atmospheric Origin of FRN and Metal Deposition

Concentrations of FRNs and metals in foliage time series reveal the interaction of atmospheric metals and PM2.5 with the forest canopy and confirm that the metals studied here are atmospheric in origin (Figure 2). Leaves were collected approximately monthly year-round, and we note that red oak retains dead leaves on the branches through autumn, winter, and into their second spring, so that the oldest (dead) leaves are sampled in their second spring at an age of about 1 year. Near-zero intercepts and monotonic increases with time in both living and dead leaves confirm an atmospheric source for FRNs and metals.^7^ Be accumulation slows in winter due to its short half-life and decreased uptake efficiency from snow versus rain.

**Figure 2:**
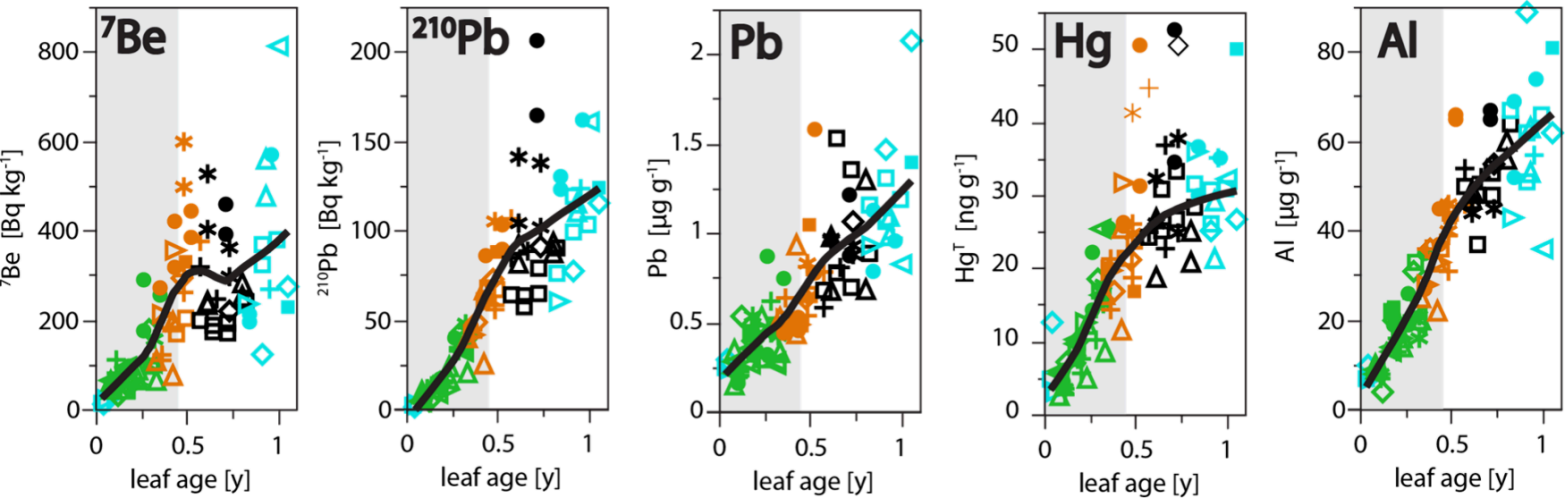
Accumulation of FRNs and metals in a timeseries of red oak foliage (*Quercus rubra*). Gray bands show the active growing season (living leaves), while white shows senescence when dead leaves are retained in their canopy positions. Symbols differentiate years of sampling (closed square = 2013, open diamond = 2014, open square = 2015, open triangle = 2016, cross = 2017, right triangle = 2018, asterisk = 2019, left triangle = 2020, closed circle = 2021). Colors indicate spring (blue), summer (green), autumn (orange), and winter (black).

### 3.2 Whole-Tree Inventories and Distributions of FRNs and Trace Metals

Measurements of FRNs and metals in whole-tree components are provided in Table S1 and illustrated in Figure S2. Distributions of the FRNs and selected metals among whole-tree compartments are illustrated in Figure 3 and tabulated in Table S2.

**Figure 3:**
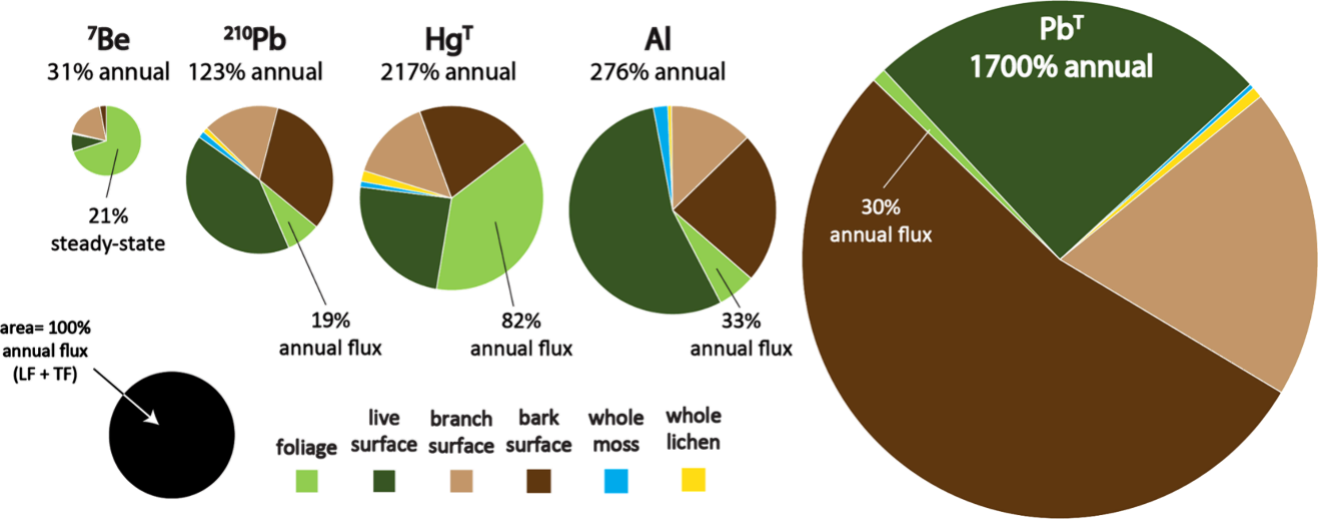
Mass distributions of atmospheric metals in the phyllosphere of the whole-tree red oak. Each pie is scaled relative to the annual atmospheric flux (black legend for scale), with a smaller ^7^Be pie showing net canopy interception and larger pies of ^210^Pb, Hg, Pb, and Al reflecting long-term accrual. The live surface is a fine mixture of moss, lichen, mold, and arboreal soil (Figure S2).

The phyllosphere accumulates metals due to its interception, absorption, and retention of wet and dry deposition. ^7^Be provides a tracer of metal interception by the forest canopy, since its short half-life (54 days) prevents it from recording long-term accrual and export from storage. Due to the importance of understanding this metric, we approached the estimation of interception in a few ways. First, the harvested whole-tree oak stores 174 Bq m^–2^ of ^7^Be, which is 39% of the steady-state inventory relative to continuous atmospheric measurements recorded at the Shattuck Observatory (20 km away; 451 Bq m^–2^).^30,34^ Interception is alternatively 31% of the total ^7^Be measured in adjacent soil following annual leaf fall (=556 Bq m^–2^). Hereafter, we assume an average of these two estimates equal to 34 ± 4%, with a corresponding uncertainty that recognizes the spatial and temporal variability in ^7^Be inventories on the landscape. Similar comparisons of ^7^Be between atmospheric deposition and local soil inventories collected in 2021–2022 show retention of ^7^Be by the canopy averaging 38.7 ± 2.7% (mean ± SE, *n* = 8) for a range of species, including eastern hemlock, red oak, sugar maple, and white pine.^35^ A third estimate of ^7^Be interception, based on openfall-throughfall comparisons for mature red oak and white pine at this site, showed that the rate of ^7^Be interception by the canopy averaged 46% as a flux-weighted mean for the two larger throughfall trees used in mass balance (below).^28^ The lower value of 34% measured in the whole-tree oak may be due to the smaller canopy of this tree, but it is consistent with the compiled average of 38.7%.

Comparing metal distributions in phyllosphere compartments provides insight into the long-term retention of metals. While the whole-tree oak intercepts 34% of wet ^7^Be deposition, approximately two-thirds of this (24% of annual deposition) is retained by foliage, and the remainder is distributed to twig and branch surfaces (6%), the whole-tree live surface (3%), and bole bark (1%). By comparison, the accumulation of ^210^Pb reflects the long-term retention of metal deposition that occurs subsequent to interception. For ^210^Pb (half-life 22.3 years), the whole-tree oak stores 123% of annual atmospheric deposition. A fraction of ^210^Pb similar to that of ^7^Be was found in foliage (19%), but the highest proportion was found in the live surface (51%) (Figure 3). Al and Pb also have foliar fractions that are similar to ^7^Be and ^210^Pb, despite much larger total inventories found throughout the whole tree. In contrast, a substantially higher foliar fraction was found for Hg^T^ (82%), which is attributable to direct uptake of gaseous elemental mercury (GEM).^39,40^ Estimated whole-tree loadings of trace metals and their percent of annual deposition (throughfall plus litterfall) are as follows: Hg^T^ = 41 μg m^–2^ (2.2-times annual); Al = 113 mg m^–2^ (2.8-times annual); Pb^T^ = 7400 μg m^–2^ (an extraordinary 17-times annual deposition) (Table S2). FRN and metal whole-tree inventories were extrapolated to the larger throughfall trees based on foliar mass and bole surface area using standard allometric equations.^38^ Average loadings extrapolated to these larger trees are as follows: ^7^Be (0.8-times steady state), ^210^Pb (11-times annual), Hg^T^ (12-times annual), Al (20-times annual), and Pb^T^ (116-times annual) (Table S2). While ^210^Pb and Pb should show similar behaviors due to their shared geochemistry, the extreme canopy loading of stable Pb is due to the historical deposition of tetraethyl Pb from gasoline combustion and underscores the fact that the canopy is not at steady-state with respect to contemporary deposition. Vast legacy Pb deposition remains stored in the canopy. For an additional perspective, the whole-tree inventories of ^210^Pb, Pb, and Hg represent approximately 17, 5.5, and 6.5% of the underlying soil inventories in organic horizons of representative local soils.

### 3.3 Autumn and Annual Litterfall Fluxes of Foliage and FPOM

Foliar litterfall in autumn is the most recognizable export of organic matter and metals from the canopy, but fine particulate organic matter (FPOM) contributes greatly to PM and metals flux in throughfall and is likely to contribute to litterfall as well.^28^ Autumn litterfall fluxes of ^7^Be and ^210^Pb for years 2020–2023 averaged 62 ± 8 and 18.5 ± 1.6 Bq m^–2^, respectively, equivalent to 11 ± 3% of ^7^Be steady-state inventory and 11 ± 2% of ^210^Pb annual flux (average ± SE, *n* = 8) (Table S3). Litterfall fluxes in autumn represent just half of the total annual litterfall flux for the FRNs; however, with a balance of leaf litter and FPOM contributions in spring, summer, and winter. Pine has a secondary cast of leaf litter in summer, and both oak and pine also cast large amounts of frass (herbivorous insect excrement) during summer that contributes to litterfall as FPOM. While FPOM contributes just 1–3% of total litterfall mass, it has the highest concentrations of ^7^Be and ^210^Pb among all terrestrial materials we have measured, including vegetation, soils, lichen, or moss, exceeding 6000 Bq kg^–1^ for ^7^Be and 2000 Bq kg^–1^ for ^210^Pb (Table S3). Despite its low mass, FPOM can contribute as much as 30% of ^210^Pb and ^7^Be annual litterfall deposition. The annual litterfall total of 120 ± 118 Bq m^–2^ for ^7^Be (*n* = 6) represents 29% of the steady-state ecosystem inventory, and for ^210^Pb the annual total of 45 ± 6 Bq m^–2^ is equivalent to 38% of annual wet + dry deposition. However, we caution that nonautumn litterfall collections may incorporate contributions from ongoing wet and dry atmospheric deposition, and more work is required to fully resolve different contributions to annual litterfall.

Autumn litterfall fluxes for Hg and Pb were 10.9 ± 1.2 and 240 ± 99 μg m^–2^ (mean ± SE, *n* = 8), respectively, and were not different between oak and pine. The autumn litterfall of Al was 29 ± 6 for pine and 6.5 ± 1.6 mg m^–2^ for oak [*p* < 0.0001]. Annual litterfall totals increased to 14.1 ± 1.5 and 551 ± 177 μg m^–2^ for Hg and Pb, respectively. The annual totals for Al were 56 ± 21 and 14.4 ± 7.7 for pine and oak, respectively.

## 4 Modeling of Canopy Mass Balance

### 4.1 Developing Canopy Mass Balances with ^7^Be and ^210^Pb

We constructed a mass balance of flows into and out of the canopy (Figure 4) using whole-tree measurements and litterfall fluxes reported here, combined with throughfall measurements from a companion paper.^28^ Specific mechanistic insights to be addressed through the canopy mass balance include (i) the absorption rate of new deposition to the canopy, (ii) the apportioning of wet and dry deposition to canopy uptake, and (iii) the exchange of long-term storage that makes the phyllosphere a source of high enrichment factors of most metals in throughfall relative to openfall precipitation. Importantly, the different half-lives of ^7^Be and ^210^Pb allow us to separate the interception of new deposition and the release of old deposition from long-term storage that has not been possible with prior efforts,^28^ e.g., Lovett and Lindberg,^41^ and to explicitly date organic matter in the phyllosphere. We stress that ^210^Pb provides a comparator with an unambiguous secondary aerosol source against which to compare other atmospheric metals such as Pb^T^, Hg^T^, and Al, which may have additional contributions from resuspended dust or, in the case of Hg, deposition from a gaseous phase,^34^ whereas ^7^Be forms the basis for understanding interception of particle-reactive PM metals by the canopy during atmospheric deposition because its short half-life prevents any contributions of legacy deposition (>1 year).

**Figure 4:**
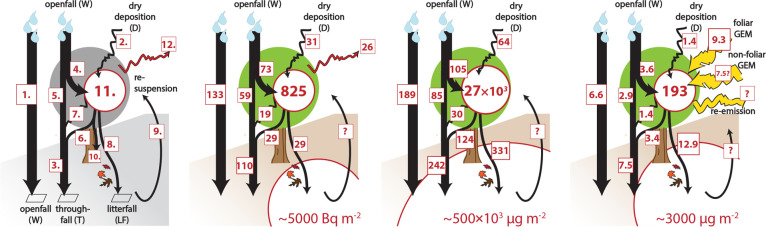
whole-tree mass balances with flows into and out of storage in the phyllosphere. (a) Processes are numbered as described in main text, (b) ^210^Pb, (c) Pb^T^, (d) Hg^T^. Boxes indicate fluxes [Bq m^–2^ year^–1^ or μg m^–2^ year^–1^] and circles indicate standing inventories [Bq m^–2^ or μg m^–2^]. The large circles at the lower right indicate the typical inventories in the organic horizon of representative soils.

The whole-tree mass balance model includes the following flows into and from the phyllosphere (Figure 4):

1. openfall deposition (*W*) was directly measured;
2. dry deposition (*D*_β_) was estimated previously by throughfall multiple-regression mass balance;^28^
3. throughfall deposition (*T*) was directly measured;
4. canopy absorption is the amount of *W* retained by the canopy (=*W–T–D*_β_ or 1–2–3);
5. direct throughfall was estimated as *W* minus absorption (1–4);
6. change in storage (Δ*S*) is the amount released from long-term canopy storage, previously estimated with FRN throughfall mass balance;^28^
7. washoff of dry deposition was estimated by closing the canopy mass balance (below);
8. litterfall deposition (LF) was directly measured;
9. resuspension or re-emission is not constrained but elsewhere estimated to be on the order of 10% of annual deposition;^34^
10. stemflow (SF) is not evaluated but likely on the order of 5% of metal budgets;^42^
11. canopy storage is the standing metal inventory from whole-tree estimates (Section 3.2);
12. radioactive decay is included as a loss process for the FRNs.

For ^7^Be, 22% of annual wet+dry deposition totaling 2080 Bq m^–2^ is attributable to dry processes. Approximately 56% of total wet+dry deposition is absorbed by the canopy on an event basis, and the remainder is transmitted as throughfall. A final 103 Bq m^–2^ or 18 ± 2% of annual deposition is removed in litterfall, and the remaining 38% persists in the canopy to be lost to radioactive decay (mean lifetime of 1/λ = 78 days). We calculate a ^7^Be absorption coefficient of 55.8% for wet deposition (=*W*/(*W*–*T*–*D*_β_), which forms the hypothesis for interception of ^210^Pb and other metals.

We can construct a preliminary mass balance for other metals using information from the ^7^Be mass balance, with a reasonable assumption that the metals are similarly particle-reactive.^28^ For ^210^Pb, total openfall deposition *W* was measured =133 ± 3 Bq m^–2^ year^–1^, dry deposition *D*_β_ = 19 ± 5 Bq m^–2^ year^–1^ was previously reported,^28^ and standing inventory in the canopy of mature oak and pine throughfall trees scaled from whole-tree measurements using allometric equations^38^ averages *S* = 825 Bq m^–2^ or 4.7 years’ worth of total deposition (Table S2). Of wet deposition, we extrapolate from ^7^Be absorption that 74 ± 2 Bq m^–2^ should be absorbed and 59 ± 6 Bq m^–2^ transmitted directly through the canopy. We measured 110 ± 2 Bq m^–2^ year^‑1^ in export of throughfall, of which we previously attributed 29 ± 2 Bq m^–2^ year^–1^ to a change in storage from the phyllosphere by throughfall mass balance.^28^ An average of 34 ± 5 Bq m^–2^ (mean ± SE) leaves the canopy by annual litterfall. The proportion of deposition exported in annual litterfall =20 ± 4% (*n* = 8), calculated as *LF*/(*W* + *D*).

Closure of the ^210^Pb mass balance can be assessed with the following three equalities: first, that (i) 4 + 5 must equal 1, which is the partitioning of new wet deposition between absorption and throughfall. Next, (ii) the throughfall mass balance of 5 + 6 + 7 should equal 3, if all contributing sources to throughfall are accounted for. Finally (iii), 4 + 5 + 2 should equal 3 + 8 as the fundamental canopy mass balance but only if all sources/sinks are fully accounted. The first equality (i) is mandated by the model. The second equality (ii) is established in the throughfall mass balance.^28^ The third equality (iii) is the mass balance of gross inputs and outputs. Here we must also include 26 Bq m^–2^ year^–1^ loss due to radioactive decay of ^210^Pb (3.1% per year of total canopy storage = 825 Bq m^–2^).

When we first assumed that there was zero absorption of dry deposition, this leaves an excess of 10 ± 6% in ^210^Pb net export from the canopy in LF + TF. The mass balance can be closed if we assume a 48 ± 11% absorption rate for dry deposition. This then requires a proportionate increase in the estimated total rate of dry deposition, *D*_T_, since this fraction bound to the canopy is invisible to throughfall measurements in deriving *D*_β_ (Figure S5). The total ^7^Be and ^210^Pb dry deposition must be increased to 410 and 37 Bq m^–2^, respectively. An absorption rate of 48% is comparable to the wet deposition absorption rate of 55% observed for ^7^Be, and similar absorption rates for wet and dry deposition are also inferred from leaf uptake of the FRNs^30^ and assumed in deriving Δ*S*.^28^ Strong absorption of dry deposition is also consistent with our prior observation that it requires up to 2 cm of rainfall to fully remove dry-deposited PM from the canopy, a precipitation amount that is exceeded only 50% of storms with a recurrence interval of about 14 days at the study site. The increased estimate of dry deposition is sufficient to maintain a steady state with respect to input and outputs of ^210^Pb, but does not account for any year-on-year accrual. Some accrual of ^210^Pb must occur, as the surface areas of the tree increase with growth. This would then, in turn, require a higher rate of absorption of dry deposition, but future work will be required to better constrain the mass balance.

### 4.2 Stable Pb Canopy Mass Balance

The contrast of ^210^Pb and Pb^T^ mass balances is important because we expect these isotopes to share elemental geochemistry, and any differences can be attributed to behaviors of different PM sources and histories of atmospheric deposition.^43,44^ For total atmospheric Pb^T^, we calculate a large net export from phyllosphere, insofar as 248 ± 66 μg m^–2^ year^–1^ enter via *W* and *D* but 669 ± 133 μg m^–2^ leave via T and LF. Our estimate that 48% of *D* is absorbed leaves just a 2% deficit in TF mass balance according to equality (ii). Still, we observe a large net export of 422 ± 149 μg m^–2^ year^–1^ (equality (iii)). While this net excess represents a majority or 63% of canopy export, it requires a loss of just 1.2% per year from storage in the phyllosphere (based on the estimated size of the reservoir and assuming a first-order loss constant). Such a low rate is consistent with essentially permanent storage of Pb, which, in turn, circles back to explain how such a large reservoir of Pb accumulates in the phyllosphere. Most of the stored atmospheric Pb^T^ will enter the soil system through coarse woody debris and tree death.

### 4.3 Mercury Canopy Mass Balance

Unlike other metals, Hg has a novel depositional process via deposition of GEM, which can be either internalized into the leaf by stomatal uptake or absorbed by the exterior of the leaf. We measured foliar uptake of GEM to be 9.1 μg m^–2^ as the measured litterfall flux (12.8 ± 1.3 μg m^–2^), minus the fraction of wet deposition estimated to sorb to foliage (3.7 μg m^–2^). GEM also might provide a novel loss process by its re-emission from prior deposition of Hg^2+^ in rainwater.^32,45^ If we include nonfoliar GEM deposition to the phyllosphere (NF) of 7.5 μg m^–2^ year^–1^ that has been inferred by both ecosystem and soil mass balances,^3,4,6^ we would observe a deficit of 21% in export of Hg from the canopy totaling −4.3 ± 1.4 μg m^–2^ year^–1^ as measured by the sum W+D-LF-TF-NF (equality (iii). This deficit would be consistent with the re-emission of GEM from the canopy.^32^ We are unable to close the Hg mass balance on the scale of an individual tree because GEM uptake is not independently constrained. However, the whole-tree mass balance allows us to make some important observations of Hg dynamics. We estimate that 3.7 μg m^–2^ year^–1^ of Hg wet deposition is absorbed by the canopy, a fraction of 56% which is close to 65% estimated in a tropical forest.^46^ Further, closure of the throughfall mass balance (equality (ii)) requires an assumption that there is no canopy absorption of GEM dry deposition. Both conclusions are consistent with those of Yuan et al.,^46^ who showed with Hg stable isotopes that a dynamic exchange occurs at the leaf surface whereby Hg^2+^ in rainfall is absorbed by foliage, whereas dry deposition of GEM to the leaf surface (a fraction not absorbed internally by the stomatal pathway) is fully rinsed off in throughfall. However, the throughfall mass balance also requires that a further 3.4 ± 0.1 μg m^–2^ year^–1^ of Hg in throughfall must be sourced from long-term storage.^28^ Thus, while the majority of Hg^2+^ is absorbed by the canopy, and even as dry deposition of GEM is efficiently washed from the foliar surface, a balance of legacy Hg is required to match the measured export in TF. Our mass balance, therefore, predicts that throughfall is a mixture of 19% GEM dry deposition, 39% direct throughfall, and 45% legacy deposition from Δ*S*. The total litterfall of 12.8 μg m^–2^ year^–1^ GEM, plus 6.6 μg m^–2^ year^–1^ wet precipitation of Hg^2+^, and 7.5 μg m^–2^ year^–1^ nonfoliar GEM yields 24.6 μg m^–2^ year^–1^ total annual Hg deposition, of which foliar GEM is 75%. This is consistent with prior studies for northeastern US forest canopies,^4,6^ but the nonfoliar GEM remains a poorly understood source to both above-ground ecosystem components as well as directly to the forest floor. We estimate that mature canopy trees store on the order of 9.9 years’ worth of historical atmospheric deposition (Table S2), which is comparable to measurements by Wang et al.^2^

### 4.4 Aluminum and Insights into the Role of PM on Metal Whole-Tree Mass Balance

Aluminum provides a metal comparator with a crustal source (primary aerosol), rather than secondary production in the atmosphere like FRNs, Pb, and Hg, and therefore provides some insight into the different mechanisms that influence PM behavior. Because of this different production mechanism, for example, the measured rate of Al dry deposition is a much higher proportion of total flux (42%) compared to the FRNs, Pb, or Hg (22–24%) (Table S2). Still, the throughfall equality (ii) is close to within 5% when we carry the same assumption of dry deposition absorption that is derived with ^210^Pb. In contrast, the canopy mass balance equality (iii) shows an excess of 29 mg m^–2^ year^–1^ or 47 ± 30% of net export that we cannot readily attribute to legacy reservoirs, as for Pb and Hg. We have no direct means to assess historical changes in the Al deposition. However, a decline in Ca deposition over the past 50 years at the Hubbard Brook Experimental Forest, a similar and well-studied forest in our region, is attributed to decreasing PM inputs to the forest via dust as clearing and road building decreased.^47^

An alternative explanation for excess Al and Pb in canopy export is leaching from metabolically active tissues of the tree. However, two arguments suggest that excess Al export derives from PM deposition. First, the increase in throughfall export is strongly associated with increases in the particulate fraction (>0.5 μm) for particle-reactive atmospheric metals, including Al as well as Be, Pb, and Hg (Figure 5a).^28^ In fact, the atmospheric deposition of crustal metals has long been attributed to resuspension of biogenic organic matter.^48^ Here we report that the fraction particulate (% > 0.5 μm) of metals in throughfall is inversely correlated with crustal enrichment, where EF_crust_ = (M_i_/Al)_TF_/(M/Al)_crust_],^28,49^ meaning that resuspended metals like Al and Fe have much larger particle sizes than secondary aerosol like the FRNs, Pb or Hg (Figure 5a). At the same time, the fraction particulate drives the enrichment (EF) of crustal metals in throughfall (EF = OF/TF fluxes), which shows that high throughfall enrichment is likely due to deposition or internal ecosystem recycling of coarse resuspended dust (Figure 5b). A second line of reasoning can be made from the accumulation of Al and Pb in the foliage (Figure 2). These metals, like other atmospheric metals, increase in foliar timeseries monotonically with near-zero intercepts at consistent rates in both live and senesced foliage, and the concentrations in year-old leaves are comparable to what we observe in litterfall (∼3 μg g^–1^ Pb and ∼100 μg g^–1^ Al). In conclusion, the suite of highly particle-reactive metals, including Al, Fe, Be, Pb, and Hg show striking patterns in their speciation and export from the canopy related to PM origin and size, which reflect a high degree of ordering in the processes regulating atmospheric deposition, residence in the phyllosphere, and transfer to soil, that impacts the biogeochemistry of PM.

**Figure 5:**
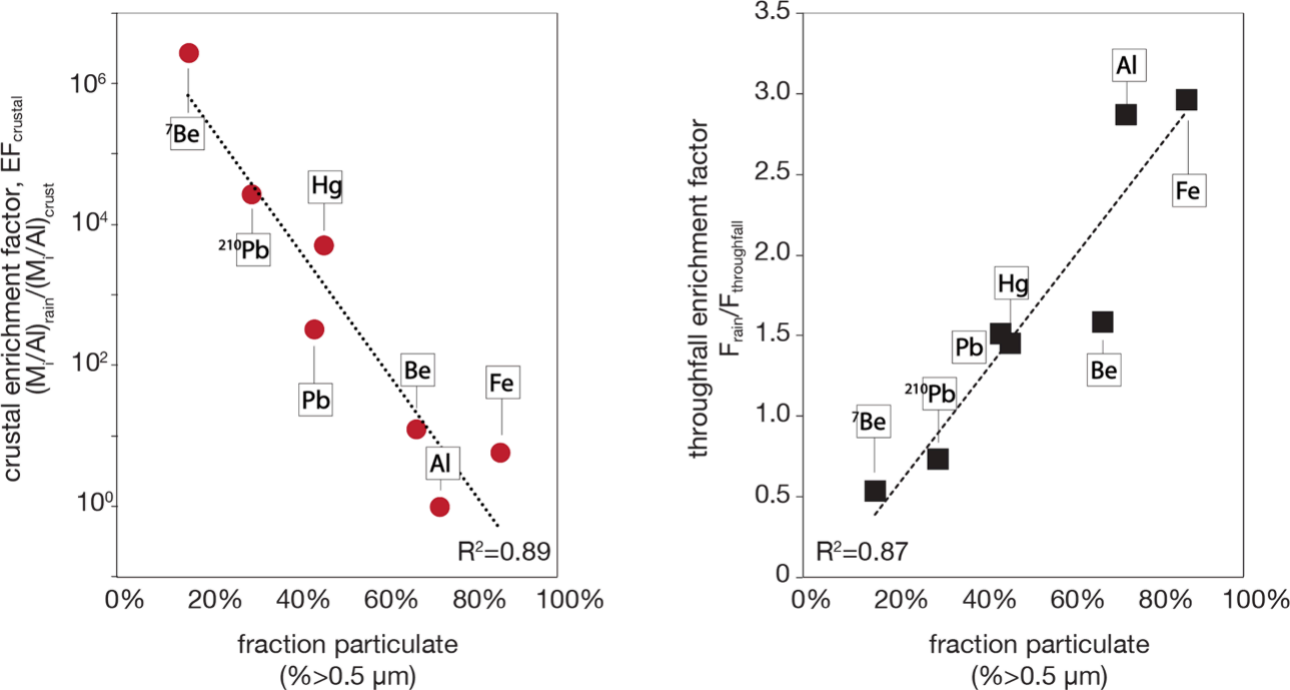
Speciation of FRNs, Hg, Pb, and Al versus export from the forest canopy. (Left) Coarsening particle size distribution or fraction particulate (% > 0.5 μm) with increasing crustal or dust source (low EF_crust_). (Right) Coarsening particle size correlates with higher throughfall enrichment (flux in rain/flux in throughfall), likely due to weathering of resuspended coarse PM metals from the phyllosphere. Data from Landis.^28^

## 5 Time Scales of Atmospheric Metal Storage in the Canopy

Understanding the time scales of storage in the phyllosphere becomes important to understanding biogeochemical cycling of metals. The age of PM in wet and dry deposition is typically in the range of 20–400 days, with dry deposition typically older due to the resuspension of dust, and the particulate fraction (>0.5 μm) consequently older than the dissolved fraction in wet deposition.^33,34^ The age of PM in the phyllosphere is exponentially older, from years to decades. We previously estimated the lifetime of ^210^Pb in the phyllosphere from the estimated reservoir (825 Bq m^–2^) and the change in storage via throughfall (29 Bq m^–2^ year^–1^), which, assuming a first-order process, gives a mean lifetime (1/*k*) in the canopy of 32 years.^28^ Ages of individual components of the phyllosphere can also be independently estimated using FRN chronometry, based on their measured ^7^Be:^210^Pb ratios (*R*) in the phyllosphere compartments and an open-system accumulation model with respect to bulk atmospheric deposition.^30^ The age estimate of foliage is important because the red oak foliage has a known age of 0.45 years based on the observed leafout date of May 1 and harvest date of 14 October. The ^7^Be:^210^Pb age of foliage measured here averages 0.42 years (Table S1). Ages of twigs and branch bark for the whole-tree oak measured here increase in the expected order similar to a law of superposition (Figure S6), from twigs (*R* = 2.7, age = 1.4 years), < 1 cm branches (*R* = 0.73, 5.5 years), 1–2 cm branches (*R* = 0.61, 6.6 years), 2–3 cm branches (*R* = 0.52, 7.9 years), and >3 cm branches (*R* = 0.50, 8.3 years). Live lichen (*R* = 0.24) was aged 21 years. Live whole moss (*R* = 0.20) was aged 28 years.

The live tree surface (*R* = 0.16) was aged 40 years on average, but decreased in age from 20 years at the crown to 51 years at the tree bottom. The bole bark at the crown (*R* = 0.27) was aged 17.8 years, but at the tree bottom, the bole bark ratio of 0.05 was lower than the steady-state ^7^Be:^210^Pb ratio of 0.11 predicted from atmospheric deposition and thus cannot be dated. Such a violation of the open-system model assumptions can result from variations in ^7^Be interception, which is a highly episodic time scale of individual rain events. Higher ^210^Pb activity at the tree base may also be attributed to dust resuspension or radon emanation from the surrounding soil. It should be emphasized that the tree was aged to 68 years by counting annual rings, which places an absolute upper limit on any FRN estimates.

## 6 Implications for Biogeochemical Cycling of Atmospheric Metals

Soils are typically the focus of investigations into the fate of atmospheric metals, since these represent the ultimate repositories for atmospheric deposition. However, the omission of above-ground storage is problematic for understanding atmospheric metal biogeochemical cycles because the phyllosphere retains large fractions of atmospheric deposition on an annual basis and consequently builds up very large reservoirs over decadal time scales. These reservoirs regulate the export of PM and associated metals from the phyllosphere and thereby control both the composition of throughfall and the introduction of metals into underlying soils. The novel use of FRNs provides an improved framework for understanding the interaction of atmospheric metals with forested ecosystems. The FRN approach quantified the partitioning of atmospheric deposition into wet and dry fractions that are absorbed and transmitted through the canopy. It also quantified contributions of new versus old metal export from the phyllosphere. Finally, FRN chronometry directly measured the ages of foliage and other biogenic materials in the phyllosphere, allowing a new perspective on the importance of metal storage to biogeochemical cycling. While the broad strokes of atmosphere-biosphere interactions in the phyllosphere are drawn here, the logistical and analytical limitations remain challenging, and our preliminary results represent just a single or a few trees. Future work to confidently scale to global forests should repeat these measurements spanning representative tree functional types, biomes, and climatic gradients, and upscaling these results by incorporating, e.g., remote sensing or photogrammetry techniques.

The export of metals from forest canopies represents a complex exchange, whereby new deposition is absorbed while old deposition is released. For Pb and Hg with long histories of industrial emissions, the phyllosphere accumulates large canopy reservoirs of legacy deposition over multidecadal time scales. The export of old deposition from these forest canopies can exceed new inputs and enrich throughfall over what would be predicted from measurements of wet and dry deposition. At the same time, the loss constant for old deposition, e.g., for Pb, is approximately 1% per year, which underscores the strength of the metal affinity for organic matter in the phyllosphere.^28^ In the absence of new FRN insights, the enrichment of metals that characterizes most throughfall fluxes would not be differentiated from new ecosystem inputs through dry deposition.^17^ Here, the combination of canopy and throughfall mass balances shows that net export of metals from phyllosphere storage is a principal contributor to throughfall metal biogeochemistry.

Central to the question of canopy metal storage and release is whether the phyllosphere can be considered at steady-state with respect to inputs and exports. For many industrial metals, we can be sure this is not the case since atmospheric deposition of, e.g., Pb and Hg, has declined in northeastern forests by factors of 10 and 2 since peaking in the 1980s–1990s.^6,50^ For crustal elements such as Al, this requires some understanding of land use, forest, and urban/rural development histories. A further complication for crustal elements, coarse PM dust resuspended from the ground, may interact less strongly with the canopy and might not be represented in event-based precipitation and throughfall collections to the same extent as FRNs and PM2.5. As a result, the observed export of Al, for example, is 44% higher than can be accounted for in event-based wet and dry deposition without invoking a legacy source of Al in the phyllosphere from, e.g., historical rural development and road building. While the FRNs provide archetypal tracers of PM2.5, improved approaches for understanding the contribution of resuspended dust to biogeochemical cycles remain necessary.

Accurate measurement of dry deposition to the forest canopy also remains a critical problem with large uncertainties. We have shown elsewhere that the multiple regression approach for measuring dry deposition of atmospheric metals is vastly more accurate than conventional throughfall mass balances,^28^ yet the multiple regression presumes that dry deposition is fully removed in rainfall.^41^ To the contrary, our observations of throughfall in our companion paper and canopy mass balance here indicate that dry deposition of cationic metals is largely retained in the canopy by some mechanism.^28,30^ We posit that this is mediated by the water of precipitation or directly from condensation of water transpired by the leaf itself.^13,51^ To the extent that dry-deposited PM metals are absorbed and retained by the canopy, the throughfall multiple-regression approach fundamentally underestimates dry flux by a proportionate degree. Here we have determined a dry absorption factor of approximately 48 ± 11% (similar to wet absorption of 55%), which closes the whole-tree annual mass balance of input and exports. The estimate of dry absorption is dependent on the mass balance for canopy exports in litterfall, and more work is required to better constrain both foliar and FPOM litter exports on a full annual basis.

Finally, discriminating atmospheric deposition from tree metabolic leachates remains an outstanding problem. All metals are likely cycled to some degree related to both the metabolic needs of the tree and elemental solubility.^13,52,53^ Certain tree species may exert uniquely strong pressure on specific elements, such as hickory on Be.^54^ Uptake of Th may vary for the same species between forest stands,^35^ and uptake of Ni may vary based on the underlying lithology.^28^ With FRN mass balances, we cannot strictly distinguish changes in storage of above-ground reservoirs from metabolic contributions through pathways internal to the tree. Future work combining metal stable isotope systems of, e.g., Pb, Hg, Cd, and Cu with FRN chronometry in throughfall should hold great promise for resolving both the pathways and time scales of biogeochemical cycling of atmospheric metals in forest canopies.

## Supplementary Material



## Notes

Images of phyllosphere components that were sampled for the whole-tree mass balance (Figures S1–S4); importance of absorption of dry deposition by the canopy and ^7^Be:^210^Pb chronometry of phyllosphere components, data produced in this study, including FRN and trace metal concentrations; inventories in phyllosphere components and litterfall; and whole-tree mass balance with FRN chronometry (PDF).
