# Supplementary material for: Storage and Export of Atmospheric Hg, Pb, Al, and Fine Particulate Matter (PM2.5) from Forest Trees
Source: Environ Sci Technol. 2025 Nov 4;59(45):24441–50. doi: 10.1021/acs.est.5c11154 (PMC12631979; doi:10.1021/acs.est.5c11154)
Supplement: Supplementary file 1 [file es5c11154_si_001.pdf]

Supplemental Information for

Storage and export of atmospheric Hg, Pb, Al, and fine particulate matter (PM2.5) from forest trees

Joshua D. Landis*

Dartmouth College, Dept. Earth Sciences, 19 Fayerweather Hill Road, Hanover NH USA 03755
*correspondence to joshua.d.landis@dartmouth.edu

This Supplemental Information consists of 9 pages, including Figures S1-S6 and Tables S1-S4.

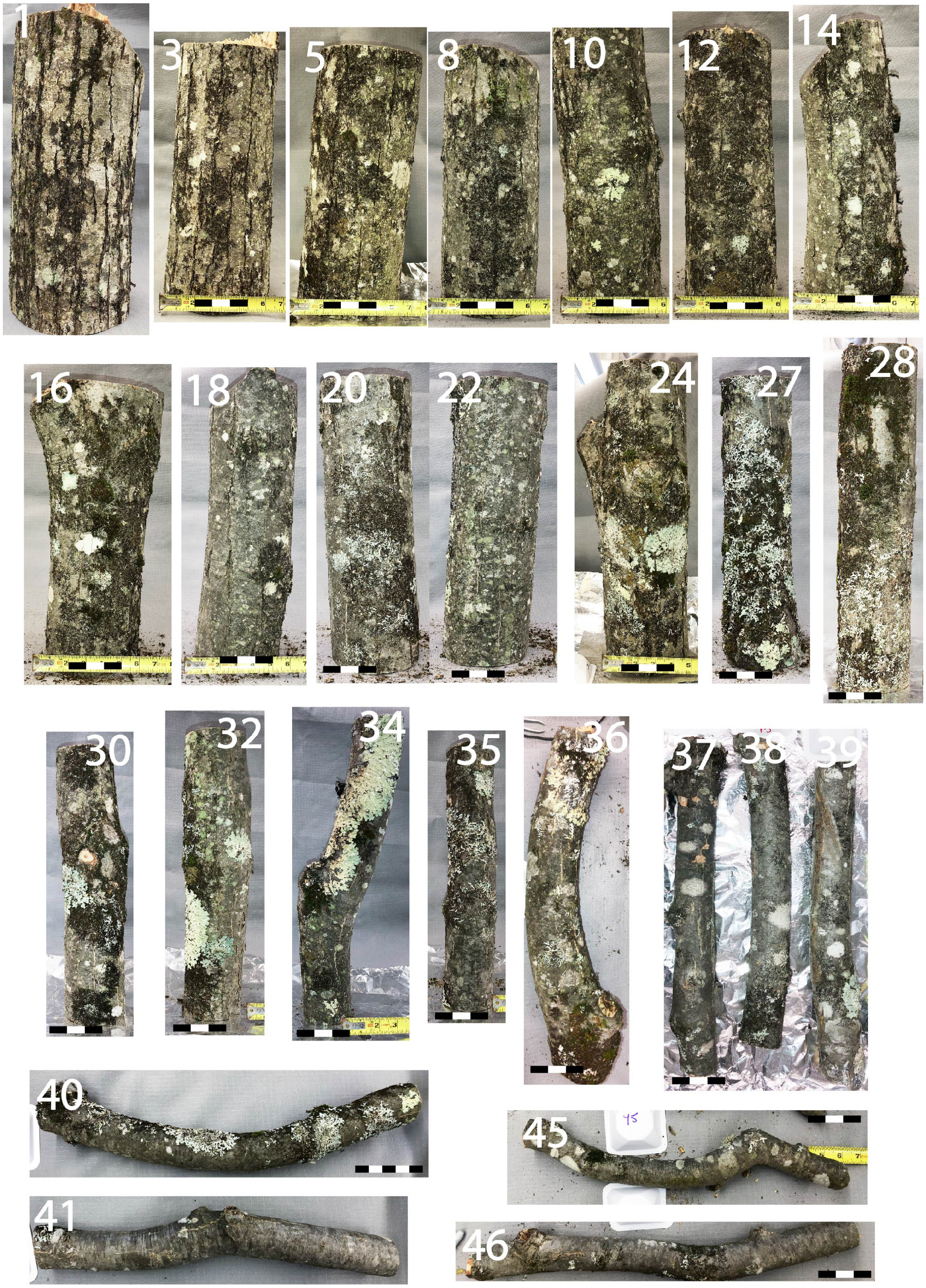

Figure S1. A selection of tree sections measured for whole tree mass balance.

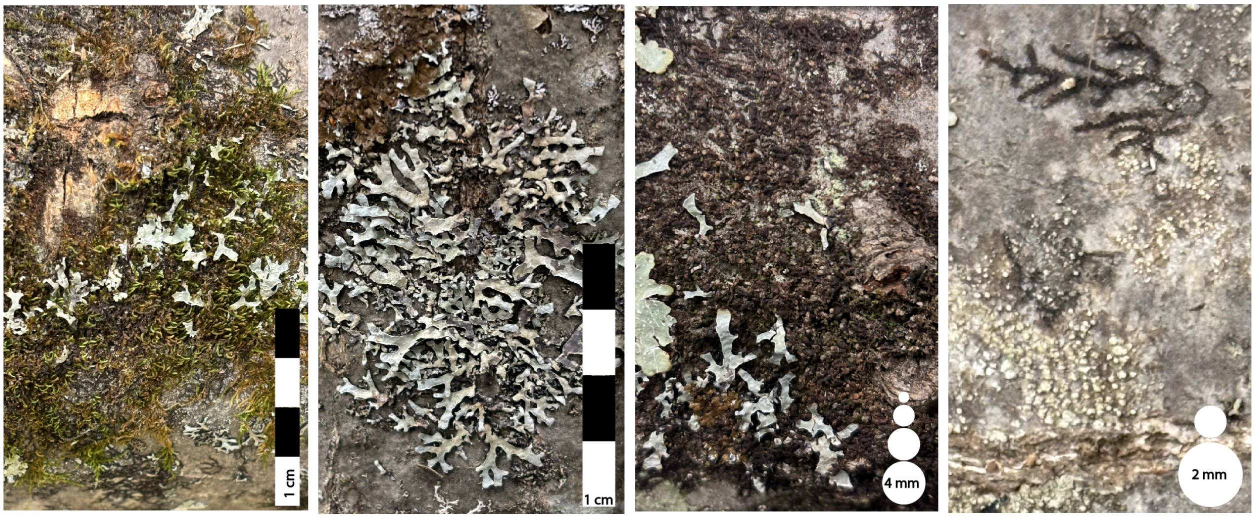

Figure S2. Components of the phyllosphere provide abundant surfaces for absorption of atmospheric metals, (a) whole moss, (b) whole fruticose lichen, (c) mixture of lichen and dead moss of the live surface, (d) crustose lichen and new moss of the live surface.

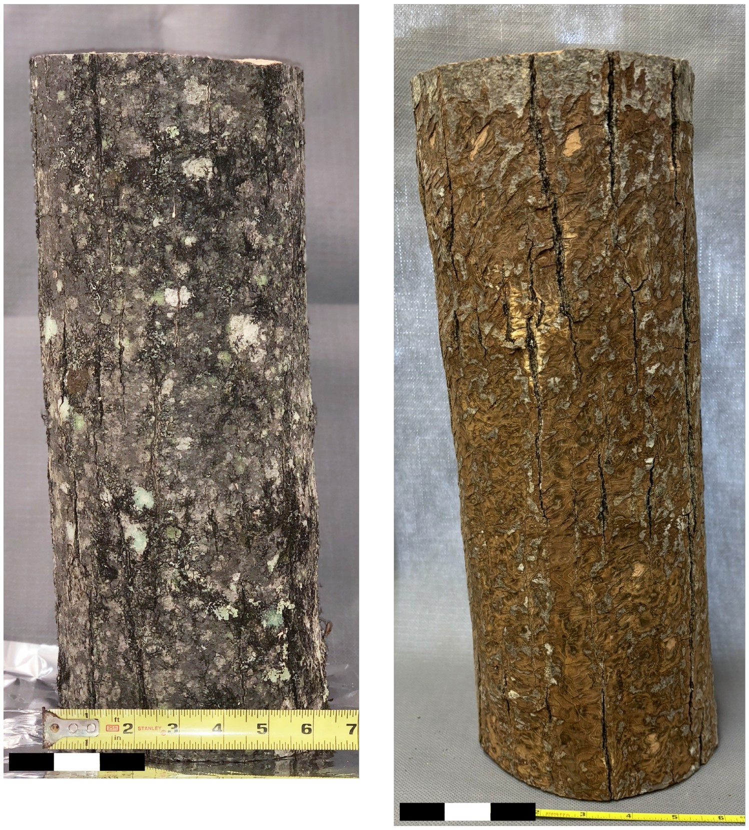

Figure S3. Before and after photos of whole-tree section #6, which was first scraped to separate the live surface of lichen, moss, and mold, and then shaved to remove the outer ca. 0.5 mm of bark.

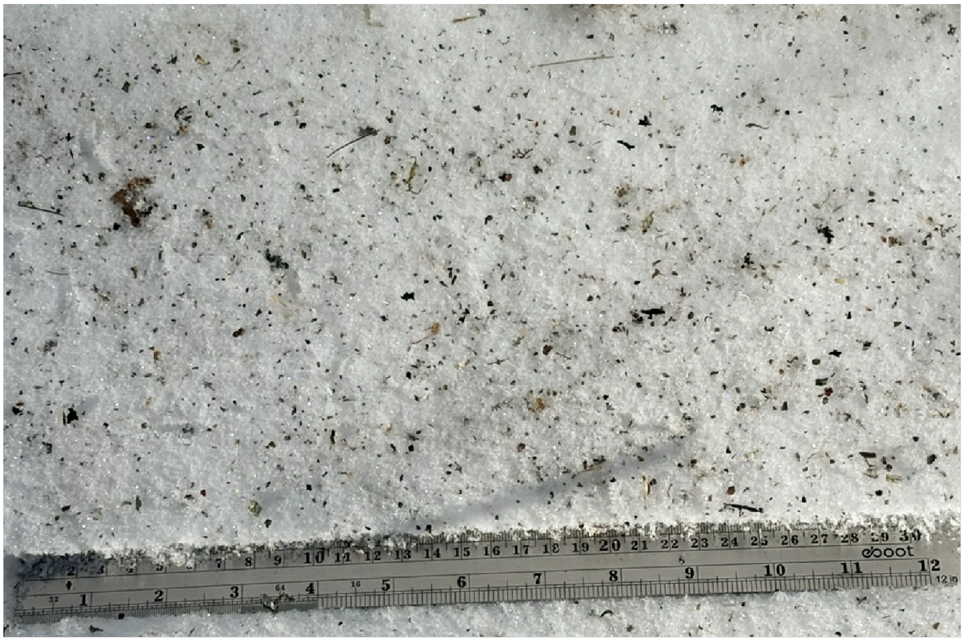

Figure S4. Accumulation of biogenic particulate organic matter (FPOM) on the snow surface. The deposition of this material is cryptic but is revealed on snow covered soils, here following a wind storm.

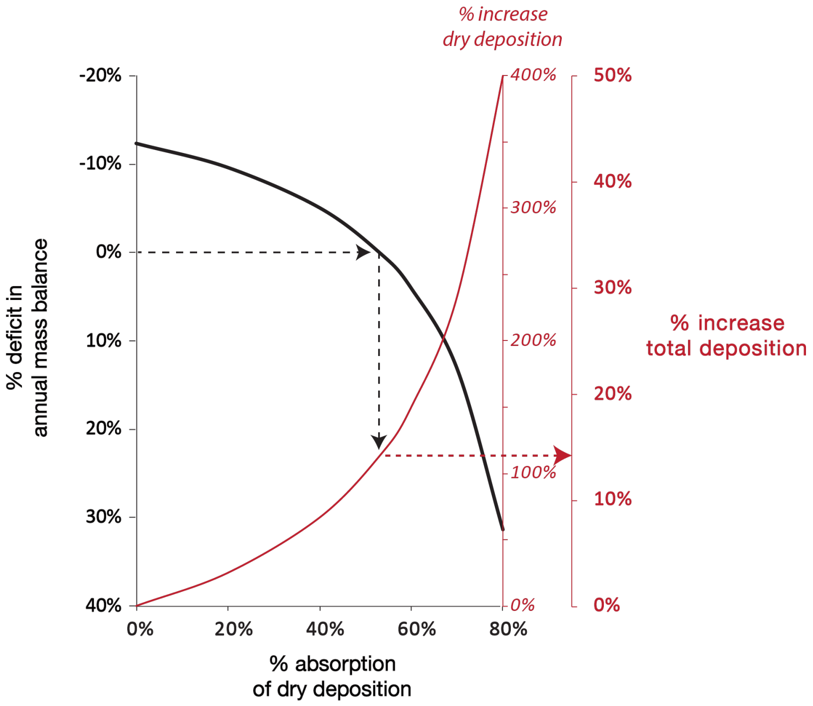

Figure S5. relationship of % absorption of dry deposition to whole-tree mass balance.
An assumed absorption of 0% leaves a 10% shortfall in ecosystem input relative to measured throughfall and litterfall exports (black line). Mass balance is closed with dry absorption of 48%, which requires a proportionate increase in the total rate of dry deposition (red line).

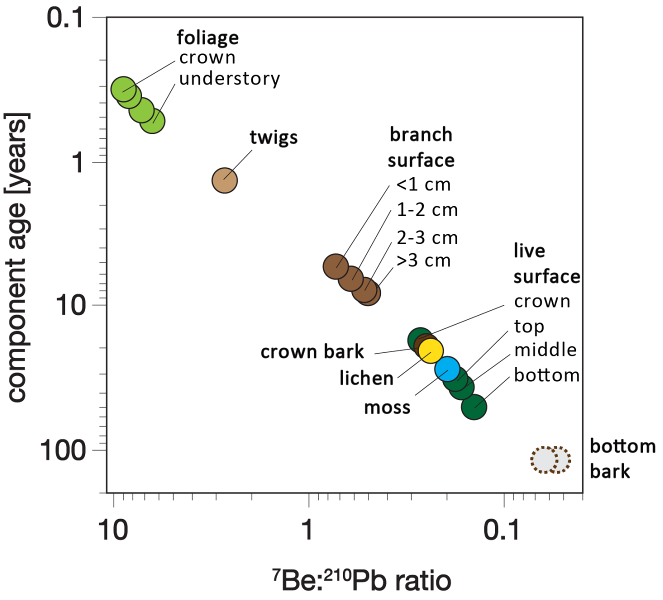

Figure S6. measured ^{7}Be:^{210}Pb ratios and equivalent age estimates of phyllosphere components.

Table S1: masses and concentrations of FRNs, Hb, Pb, and Al in whole tree compartments

compartment description	*sample #*	**compartment name**	**dry mass g m^{-2}**	**^{7}Be Bq kg^{-1}**	**^{210}Pb Bq kg^{-1}**	**Hg^{T} ng g^{-1}**	**Pb^{T} µg g^{-1}**	**Al µg g^{-1}**
leaves	*w.t 01*	tier 1	135	299	36	29.5	0.176	16.0
	w.t 02	tier 2	82	286	32	28.5	0.126	10.7
	w.t 03	tier 3	80	228	32	30.9	0.173	15.0
	w.t 04	tier 4	76	188	30	*32*	0.142	14.8
	w.t 05	tier 5	128	194	31	33.9	0.052	10.8
twig surface	*w.t 06*	1-3 nodes	208	67	25	6.6	1.0	9.5
branch surface	*w.t 07*	<1 cm	62	67	92	16.4	2.6	28.5
	w.t 08	1-2 cm	62	*65*	*106*	*20.3*	1.8	49.2
	w.t 09	2-3 cm	68	62	120	16.2	4.1	50.2
	w.t 10	>3 cm	71	71	143	17.4	9.5	62.6
whole moss	*w.t 11*	#1-27	4	150	769	99.0	5.1	609.1
live surface	*w.t 13*	bottom (1-15)	52	81	569	87.6	25.6	466.9
	w.t 15	middle (16-30)	46	126	765	75.8	9.0	572.5
	w.t 18	top (31-40)	19	185	1120	92.9	5.9	520.4
	w.t 20	crown (40-45)	3	306	1226	97.6	6.6	635.3
outermost bark	*w.t 14*	bottom (1-15)	227	7	123	18.3	10.1	50.4
	*	middle (15-30)	189	*10*	*166*	*16*	*4*	*61*
	*	top (30-40)	72	*16*	*90*	*12*	*10*	*34*
	w.t 19	crown (40-45)	27	26	98	12.6	10.9	41.8
whole lichen	*w.t 17*	whole lichen	6	92	386	128.4	8.5	104.9

** extrapolated from #14, #19*

Table S2: whole-tree inventories of FRNs beryllium-7 and lead-210, and Pb, Hg and Al.

	beryllium-7 [Bq m^{-2}]			lead-210 [Bq m^{-2}]			total Pb [µg m^{-2}]			total Hg [µg m^{-2}]			aluminum [mg m^{-2}]		
	w.t. oak	TF oak	TF pine	*w.t. oak*	TF oak	TF pine	w.t. oak	TF oak	TF pine	*w.t. oak*	TF oak	TF pine	*w.t. oak*	TF oak	TF pine
dbh [cm]	16	28	59	16	28	59	16	28	59	16	28	59	16	28	59
LAI [m^2 m^{-2}]	3.0	3.1	2.8	3.0	3.1	2.8	3.0	3.1	2.8	3.0	3.1	2.8	3.0	3.1	2.8
annual total*	565			155			434			19.4			41.0		
crown area (m^2)	3.1	4.9	9.6	3.14	4.91	9.62	3.14	4.91	9.62	3.14	4.91	9.62	3.14	4.91	9.62
annual eq.	31%	46%	63%	138%	357%	707%	1705%	4449%	8024%	212%	517%	1474%	276%	712%	1382%
whole tree	174	260	356	215	553	1097	7399	19310	34830	41	100	286	113	292	566
foliage	121	125	113	16	35	173	65	140	698	16	33	167	7	14	72
twig	14	36	66	89	232	413	1862	4867	8666	10	26	47	62	161	288
branch	0.62	1.5	2.9	3	8	15	21	55	97	0	1	2	3	7	12
live moss	0.55	1.4	2.6	2	6	11	51	134	239	1	2	4	1	2	3
live surface	31	82	146	36	94	167	1435	3750	6677	6	16	28	15	38	68
bole (bark)	5	14	25	68	179	319	3964	10364	18452	8	22	39	27	70	124

*for ^{7}Be we provide the steady-state equivalent flux of annual deposition due to its short half-life

Table S3: litterfall mass and metal fluxes

						total deposition							dry weight concentrations									
						dry mass	Hg^T	Al	Pb^T	^{210}Pb	^{7}Be		Hg^T		^{210}Pb		^{7}Be		Al		Pb^T	
sample	deploy	collect	season	description	m^{-2}	$g\ m^{-2}$	$ug\ m^{-2}$	$mg\ m^{-2}$	$ug\ m^{-2}$	$Bq\ m^{-2}$	$Bq\ m^{-2}$		$ng\ g^{-1}$	σ	$Bq\ kg^{-1}$	σ	$Bq\ kg^{-1}$	σ	$ug\ g^{-1}$	σ	$ug\ g^{-1}$	σ
LF-001	10/7/20	12/1/20	*autumn*	pine needles	0.22	128	3.5	23.2	188.9	7	15		27.5	4.9	53.6	6.0	117	14	181.9	15.0	1.48	0.15
LF-002	10/7/20	12/1/20	*autumn*	mixed	0.22	226	8.7	7.5	171.3	15	55		38.7	0.8	68.0	4.5	244	13	33.4	2.7	0.76	0.14
LF-003	10/7/20	12/1/20	*autumn*	broad leaf	0.44	363	11.9	6.9	233.8	24	69		32.7	0.8	66.3	5.1	189	16	18.9	1.6	0.64	0.14
LF-007	9/14/21	10/1/21	*autumn*	pine needles	10.03	20.1	0.4	3.8	9.7	1.1	4.0		22.3		55.2	7.1	198	14	187.5	15.4	0.48	0.14
LF-008	9/14/21	10/1/21	*autumn*	mixed	10.03	20.8	0.7	0.6	11.3	1.2	6.1		35.5		60.0	6.1	291	13	27.2	2.2	0.54	0.15
LF-009	9/14/21	10/1/21	*autumn*	mixed	10.03	5.6	0.2	0.7	16.3	0.8	2.4		29.4		143.4	6.9	426	16	130.4	10.9	2.90	0.76
LF-010	10/1/21	10/10/21	*autumn*	pine needles	10.03	34	0.9	7.1	25.2	2.5	6.6		26.8		73.0	6.5	192	11	204.6	16.8	0.73	0.41
LF-011	10/1/21	10/10/21	*autumn*	mixed	10.03	35	1.0	0.4	17.9	1.9	10.3		29.3		55.6	5.5	297	13	12.8	1.1	0.51	0.15
LF-012	10/10/21	10/16/21	*autumn*	pine needles	10.03	53	1.3	11.9	38.2	3.4	8.6		25.3		64.4	6.1	163	11	225.5	18.5	0.72	0.13
LF-013	10/10/21	10/16/21	*autumn*	mixed	10.03	52	2.1	4.3	45.4	3.9	18.2		39.7		75.4	6.4	348	14	83.0	6.8	0.87	0.15
LF-014	10/16/21	4/30/22	*over winter*	pine needles	10.03	11	0.4	0.0	0.0	1.2	2.6		38.0		112.3	0.7	240	1				
LF-015	10/16/21	4/30/22	*over winter*	mixed	10.03	25	0.6	0.0	0.0	9.0	25.4		24.4		352.5	1.7	997	4				
LF-016	10/16/21	4/30/22	*over winter*	mixed	10.03	28	1.4	7.9	66.9	10.5	20.7		50.8	3.3	369.6		731		280.3	23.0	2.36	0.14
LF-017	9/14/21	10/1/21	*autumn*	broad leaf	6.32	60	1.7	0.8	29.1	3.6	17.0		28.2		60.1	6.3	284	11	13.4	1.1	0.48	0.14
LF-018	10/1/21	10/10/21	*autumn*	broad leaf	6.32	108	4.1	2.4	44.5	6.6	37.1		37.5		61.0	6.6	342	15	21.8	1.8	0.41	0.14
LF-019	10/10/21	10/16/21	*autumn*	broad leaf	6.32	221	6.9	3.7	122.1	11.5	59.3		31.2		52.0	6.0	269	12	16.8	1.4	0.55	0.15
LF-020	10/16/21	4/30/22	*over winter*	broad leaf	6.32	46	2.1	6.6	59.8	13.4	33.5		46.4	4.9	291.5		728		144.1	11.8	1.30	0.14
LF-025	4/30/22	10/1/22	*over summer*	pine needles	10.03	202	5.5	41.6	730.7	27.4	52.5		27.2	1.2	135.6	0.5	259.8	1.4	205.7	16.9	3.62	0.41
LF-027	10/1/22	10/15/22	*autumn*	pine needles	10.03	175	7.2	12.1	177.5	9.0	28.4		41.4	1.8	51.3	0.4	162.2	1.2	69.4	5.7	1.01	0.15
LF-029	10/15/22	10/30/22	*autumn*	pine needles	10.03	77	3.2	5.0	69.0	7.8	24.6		41.3	1.4	100.5	0.6	318	2	65.0	5.3	0.89	0.15
LF-031	10/30/22	4/15/23	*over winter*	mixed	10.03	33	1.8	12.0	108.8	11.1	26.0		55.6		336.5	3.7	790.4	7.7	365.2	29.9	3.30	0.14
LF-032	10/30/22	4/15/23	*over winter*	pine FPOM	10.03	3.3	0.3	7.0	34.6	7.0	22.3	15%	99.7		2116.8	10.6	6780.2	24.9	2137.4	175.3	10.51	0.22
LF-026	4/30/22	10/1/22	*over summer*	broad leaf	6.32	87	1.6	6.1	71.6	11.8	23.8		18.3	0.6	134.7	0.7	272.6	1.8	69.6	5.7	0.82	0.14
LF-028	10/1/22	10/15/22	*autumn*	broad leaf	6.32	263	9.8	4.3	221.5	15.6	38.9		37.5	1.8	59.4	0.5	148.1	1.1	16.5	1.4	0.84	0.14
LF-030	10/15/22	10/30/22	*autumn*	broad leaf	6.32	137	5.1	5.0	179.9	7.1	22.3		36.8	0.5	51.3	0.4	162	1	36.3	3.0	1.31	0.14
LF-033	10/30/22	4/15/23	*over winter*	oak FPOM	6.32	14	0.6	7.1	78.5	15.1	48.9	37%	43.0		1110.0	7.4	3588.9	21.3	519.5	43.0	5.76	0.21
LF-034	4/15/23	9/10/23	*over summer*	pine needles	10.03	110	3.4	23.9	142.0	16.3	34.9		31.1		147.7	0.5	316.5	1.4	217.2	17.8	1.29	0.15
LF-035	4/15/23	9/10/23	*over summer*	FPOM	10.03	5	0.3	2.1	11.0	1.9	3.6		71.1		412.3	4.7	794.6	9.6	467.3	38.3	2.41	0.14
LF-036	9/10/23	10/20/23	*autumn*	pine needles	10.03	239	9.6	28.2	763.1	16.9	43.3		40.3		70.9	0.5	181.5	1.9	118.1	9.7	3.20	0.14
LF-037	10/20/23	4/20/24	*over winter*	pine needles	10.03	44				7.2	13.9				162.3	1.0	315.8	16.1				
LF-040	10/20/23	4/20/24	*over winter*	FPOM	10.03	2.5				2.3	4.2				927.2	12.1	1714.8	172.8				
LF-038	4/15/23	9/10/23	*over summer*	BM oak	6.32	43	1.2	1.4	85.9	14.2	42.3		27.0		326.1	1.2	972.7	4.2	31.7	2.6	1.98	0.14
LF-039	9/10/23	10/20/23	*autumn*	BM oak	6.32	334	10.9	5.8	216.1	19.0	62.9		32.6		56.9	1.7	188	5	17.4	1.4	0.65	0.14
LF-041	10/20/23	4/20/24	*over winter*	BM oak	6.32	18	0.0															
LF-042	10/20/23	4/20/24	*over winter*	FPOM	6.32	0.7	0.0															

Table S4 : whole tree mass balance for FRNs, Hg, Pb, and Al (for Beaver Meadow site)

mass balance step #		dry washoff ^{7}Be Bq m^{-2} y^{-1}	coeff.	σ	dry absorbs ^{7}Be Bq m^{-2} y^{-1}	σ	^{210}Pb Bq m^{-2} y^{-1}	σ	Pb^{T} ug m^{-2} y^{-1}	σ	Hg^{T} ug m^{-2} y^{-1}	σ	Al t. mg m^{-2} y^{-1}	σ	carbon g m^{-2} y^{-1}		note
	total deposition	*1860*			*2056*	*63*	*170*	*14.3*	*248*	*66*	*7.96*	*0.64*	*36*	*6*			*sum of wet and dry inputs*
1	**W**	1647			1647	33	133.2	5	189	6	6.57	0.39	21.2	0.4			*measured*
	model dry, Dβ	213			213	53	19.0	5.3	30.3	34	1.40	0.51	8.0	2.9			*modeled as beta in throughfall multiple regression (ref. 27)*
2	total dry, D				410	53	36.5	13.3	58	66	1.40	0.51	15.3	5.5			*estimated as measured divided by washoff coefficient*
	dry %	*11%*			*20%*		*22%*		*24%*		*18%*		*42%*				*% total annual*
3	**T**	940			940	*19*	109.9	*4.5*	242	*8*	7.48	*0.45*	39.1	*0.8*	23.5	0.7	*measured*
	**EF**	57%			57%	2%	83%	5%	128%	6%	114%	10%	185%	5%			*observed enrichment factor (EF=OF/TF)*
4	*W absorption*	*919*	*55.8%*	*3.6%*	*919*	*18*	*74.3*	*3.1*	*105.7*	*3.3*	*3.7*	*0.2*	*11.8*	*0.2*			*absorption (does not include dry)*
5	*direct T*	*727*	*44.2%*	*3.5%*	*727*	*56*	*58.8*	*7*	*83.6*	*35*	*2.9*	*0.7*	*9.3*	*3*			*direct throughfall from ^{7}Be, does not include dry*
6	**ΔS**	***0***			*0*		29.3	1.2	124.2	3.9	3.35	0.1	23.8	0.2			*detailed in ref. 27*
	ΔS % export						*17.3%*		*18.6%*		*14.3%*		*34.6%*				*ΔS fraction net export*
7	***dry washoff***	*213*	*52%*	*11%*	*213*	*53*	*19.0*	*8*	*30.3*	*35*	*1.40*	*0.6*	*8.0*	*3.3*			*adjust washoff coefficient to minimize TF mass balance error*
	dry absrption		*48%*	*11%*	*196.6*		*17.5*		*28.0*		*0.0*		*7.3*				*estimate to close mass balance*
8	**LF**	102.6			102.6	13	34.1	5.6	427	133	15.9	1.3	30	9.5	209	14	*whole-year litterfall*
	LF % input	6%			18%	2%	20%		172%		200%		81%				*% annual gross input; ^{7}Be is % steady-state (stored)*
10	***SF***																
	foliar GEM										*12.3*						*Hg only*
	non-foliar GEM										*7.5*						*approximated from Obrist et al. (ref 3) and Zhou et al. (ref 4)*
9	**resuspension**						17.0		24.8		0.80		*4.3*				*assuming 10% (below)*
	resuspension %						*10%*		*10%*		*10%*		*10%*				*difference of dry and bulk deposition rates, Landis et al. 2021 (ref)*
11	**tree storage**	308			308		825		27070		193		429				*measured/extrapolated from whole tree by allometric equations*
	storage factor	0.56			0.56		*4.9*		*109*		*9.2*		*11.8*				*years' deposition in storage*
12	**decay**						25.6										*1st order decay of storage (^{210}Pb only)*
	**canopy absorption**						92		134		3.7		19				*total wet+dry absorbed by canopy*
	total absorption %						*54.1%*		*54.0%*		*55.8%*		*52.5%*				*%wet + dry absorbed*
**total input**	***1+2***	1860			2056	63	170	14	248	66	27.7	0.6	36	6			
**total export**	***3+7***	1043			1043	23	170	7	669	133	23.4	1.4	69	10			
	mass balance equalities																
	4+5	1647			1647	59	133.2	7.6	189	35	6.57	0.71	21.2	3.0			*partitioning of wet and dry to direct throughfall*
	wet deposition (1)	*1647*			*1647*		*133.2*		*189*		*6.57*		*21.2*				
	(i) % difference	***0.0%***			***0.0%***		***0.0%***		***0.0%***		***0.0%***		***0.0%***				
	**5+6+7**	**940.4**			**940**	**77**	**107**	**11**	**238**	**50**	**7.6**	**0.9**	**41.1**	**4.5**			*partitioning throughfall contributions*
	TF export (3)	*940.4*			*940*	*19*	*109.9*	*4.5*	*242.5*	*7.6*	*7.5*	*0.4*	*39.1*	*0.8*			
	(ii) % difference	***0.0%***			***0.0%***	***8%***	***2.6%***	***11%***	***1.8%***	***21%***	***-2.2%***	***13%***	***-5.1%***	***11%***			
	**1+2**	1860			2056		170	7.6	248	66.5	27.73	0.6	36	5.5			*annual mass balance*
	LF+TF export	*1043*			*1043*		*144.0*	*7.2*	*669*	*133.3*	*23.4*	*1.4*	*68.7*	*9.5*			
	with decay						*169.7*		*x*		*x*		*x*				
	(iii) excess in export						*-0.1*	*10.5*	*422*	*149.0*	*-4.3*	*1.5*	*32.3*	*11.0*			*positive is shortfall in inputs that must be explained elsewhere*
	including decay						***0.0%***	***6.2%***	***63%***	***22%***	***-18%***	***5%***	***47%***	***30%***			
							constrained to zero		Δ storage, resuspension		GEM re-emission		Δ storage, resuspension				
